# Cooperative Jamming-Based Physical-Layer Group Secret and Private Key Generation

**DOI:** 10.3390/e26090758

**Published:** 2024-09-04

**Authors:** Shiming Fu, Tong Ling, Jun Yang, Yong Li

**Affiliations:** 1 School of Artificial Intelligence, Chongqing University of Education, Chongqing 400065, China; fusm@cque.edu.cn; 2 School of Communications and Information Engineering, Chongqing University of Posts and Telecommunications, Chongqing 400065, China; lingt2023@163.com (T.L.); s210101162@stu.cqupt.edu.cn (J.Y.); 3 College of Computer Science, Chongqing University, Chongqing 400044, China

**Keywords:** physical layer security, group key generation, secret key, private key, key capacity

## Abstract

This paper explores physical layer group key generation in wireless relay networks with a star topology. In this setup, the relay node plays the role of either a trusted or untrusted central node, while one legitimate node (Alice) acts as the reference node. The channel between the relay and Alice serves as the reference channel. To enhance security during the channel measurement stage, a cooperative jamming-based scheme is proposed in this paper. This scheme allows the relay to obtain superimposed channel observations from both the reference channel and other relay channels. Then, a public discussion is utilized to enable all nodes to obtain estimates of the reference channel. Subsequently, the legitimate nodes can agree on a secret key (SK) that remains secret from the eavesdropper (Eve), or a private key (PK) that needs to be secret from both the relay and Eve. This paper also derives the lower and upper bounds of the SK/PK capacity. Notably, it demonstrates that there exists only a small constant difference between the SK/PK upper and lower bounds in the high signal-to-noise ratio (SNR) regime. Simulation results confirm the effectiveness of the proposed scheme for ensuring security and efficiency of group key generation.

## 1. Introduction

With the widespread application of wireless devices, the demand for information sharing and communication among multiple devices is increasing, making ensuring information security especially crucial. Physical layer key generation (PLKG), based on information theory, has gained extensive attention as an effective approach for safeguarding wireless communication [1,2,3,4,5,6,7,8]. Most of the existing studies focus on PLKG for two legitimate users. However, the generation of a group key for secure group communications is essential in various scenarios, such as corporate meetings, military operations, healthcare teams, family or personal groups, and more. For example, in a business environment, a team of employees working on a confidential project may require secure communication. The generation of a group key ensures that only authorized team members can access sensitive information and discussions. Military units often rely on secure communication channels to share strategic information, where a group key guarantees that only authorized personnel can access and exchange mission-critical data. Medical professionals engaged in patient care or research may need a secure communication channel to exchange patient data and medical information, ensuring privacy and compliance with regulations. Similarly, a group of travelers may wish to share travel plans and photos exclusively within their group, ensuring that this information remains private and inaccessible to individuals outside the group [9].

Compared to the key generation involving just two parties, the key generation among multiple terminals faces more challenges. These challenges primarily stem from two main reasons. First, due to the large number of users in the group and varying distances between them, the efficiency of protocols for secure group communication is of greater concern. Second, the presence of numerous random channels among group users adds complexity to ensure that only authorized users within the group can obtain the final group key. Ref. [10] first studied the problem of group key generation for multiple users, considering a scenario where a group of users generate keys shared by all users within the group with the assistance of other users. Since then, refs. [11,12,13,14] investigated pairwise key generation schemes to generate keys shared within the group. These schemes first generate pairwise keys between two users and then generate the final group key by exploiting these pairwise keys. Additionally, ref. [15] studied the collaborative secret key generation scheme based on received signal strength, applicable to both star and chain network typologies. Ref. [16] investigated the cooperative group key generation based on secure network coding (SNC) via star topology. Moreover, ref. [17] introduced an efficient scheme for group key generation utilizing multiple-input multiple-output (MIMO) technology, which can improve the overall efficiency of key generation.

Most of the studies mentioned above have focused on the generation of keys with a single security level [9,11,12,13,14,15,16,17]. However, in practice, the generation of keys with different security levels is also important. For example, in a group of terminals, each terminal may have a different security clearance level, and access to confidential information is based on individual clearance levels. Terminals with the same clearance level should share a common key while remaining unaware of keys at higher levels. Several studies have addressed the generation of multiple keys with different security levels [10,18,19,20,21,22,23,24]. Refs. [18,19,20] investigated schemes for generating a secret key (SK) and a private key (PK) among three terminals. In these schemes, the three terminals aim to share an SK that remains secret from an eavesdropper (Eve), while two legitimate terminals aim to generate a PK that is also concealed from the third terminal. Since then, refs. [22,23,24] extended the three-terminal model to a four-terminal model with the assistance of helper nodes. These studies explored the issue of generating two keys with different security levels over a four-terminal system.

In large-scale dynamic wireless networks, it is crucial to consider both the efficiency and security in group key generation to ensure the reliable implementation of secure group communication. However, most existing schemes are based on pairwise key generation [11,12,13,14,22], which involves multiple key agreements and results in a low efficiency in key generation. To address this issue, ref. [16] proposed a secure network coding (SNC)-based group key generation scheme that requires only one key agreement phase. However, this scheme did not utilize external relay nodes to enhance the key rates, and only one type of keys was generated. Motivated by this, this paper explores physical layer group key generation in wireless relay networks with a star topology. In this setup, the relay node can act as either a trusted or untrusted central node, while one legitimate node (Alice) serves as the reference node. The channel between Alice and the relay node is considered as the reference channel. The main contributions are summarized as follows:

- We propose a cooperative jamming (CJ)-based group key generation scheme. This scheme allows the relay to obtain superimposed channel observations from both the reference channel and other relay channels. Then, a public discussion is utilized to enable all nodes to obtain estimates of the reference channel. Subsequently, either an SK or a PK is generated among the group of legitimate nodes, where the PK is secret from both the relay and Eve. This scheme only requires two rounds of key agreement regardless of the increase in the number of users within the group.
- We derive the lower and upper bounds on the SK/PK capacity within the framework of the corresponding discrete memoryless source (DMS) models. The lower bounds are determined by exploiting the zero forcing (ZF) method. Additionally, enhanced DMS models are constructed to obtain the upper bounds.
- We demonstrate that the derived lower bounds are close to the corresponding upper bounds. Specifically, at a high signal-to-noise ratio (SNR), for both the SK and PK, there is merely a constant difference of $\log_{2}\left(\frac{3}{2}\right)$ bits per channel measurement (BPCM) separating the upper and lower bounds. This difference is negligible as the transmit SNR approaches infinity.

## 2. System Model

**Figure 1:**
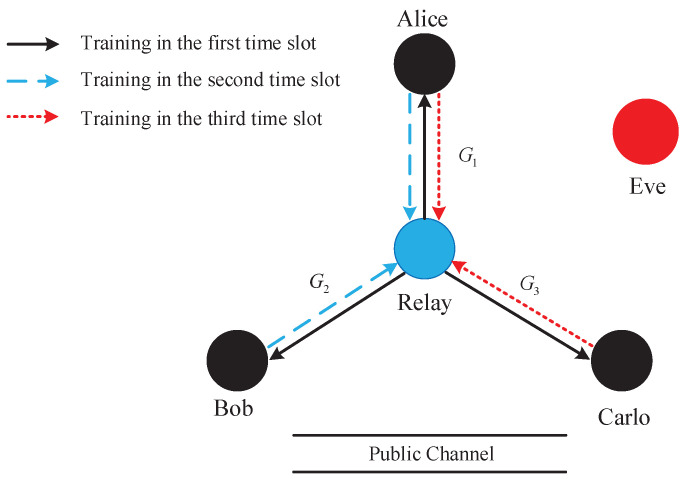
The model of group key generation and the process of training.

As shown in Figure 1, for the sake of brevity, a group communication network architecture is considered, which consists of three legitimate nodes (As shown later in this paper, the proposed group key generation scheme can be applied to the models with arbitrary number of legitimated nodes in a straightforward manner.): Alice, Bob, and Carlo, along with a relay node and an Eve. Each of these nodes has a single antenna and functions in a time-division duplex (TDD) mode of operation. The relay node can act as either a trusted or untrusted central node. We assume that Eve can intercept information and estimate the channels but cannot interfere with the communication channels or alter any transmitted messages. Moreover, to maintain anonymity, Eve positions himself more than half a wavelength away from group members, leading to the eavesdropping channels remaining independent from the legitimate channels. According to the channel reciprocity, the channel gains between the relay and Alice, Bob, and Carlo are $G_{1}$, $G_{2}$, and $G_{3}$, respectively. Note that channel gains are also used to represent the respective channels, provided that this does not cause any confusion. Moreover, it is assumed that the channels among legitimate nodes cannot be utilized for key generation due to lacking randomness. Key generation relies on the relay channels $G_{1}$, $G_{2}$, and $G_{3}$. Moreover, Alice serves as the reference node. Without loss of generality, the channel between Alice and the relay node, i.e., $G_{1}$, is considered as the reference channel (To generate common randomness, the reference channel, $G_{1}$, needs to be estimated by all legitimate nodes. It is worth noting that any relay channel can be chosen as the reference channel.).

A typical application scenario is Unmanned Aerial Vehicle (UAV) communication, where Alice, Bob, and Carlo are UAVs, and the relay is a ground base station or control console. Since UAVs operate in the air, only line-of-sight (LoS) channels exist among them. However, the channels between the UAVs and the ground base station or control console include non-line-of-sight (NLoS) components; hence, there exists randomness that is suitable for key generation. For simplicity, the wireless channels are assumed to be Rayleigh (The proposed group key generation scheme can also be simply applied to the models with Rician channel assumption, by exploiting the NLoS components.) block fading channels, where the channel gains remain constant during the coherence time. Furthermore, $G_{1}∼\mathrm{CN}\left(0,p_{A}\right)$, $G_{2}∼\mathrm{CN}\left(0,p_{B}\right)$, and $G_{3}∼\mathrm{CN}\left(0,p_{C}\right)$, where $p_{A}$, $p_{B}$, and $p_{C}$ represent the variances of the channel gains $G_{1}$, $G_{2}$, and $G_{3}$, respectively. In addition to wireless channels, a noiseless and infinite-capacity public channel where the group members communicate with each other is also considered. The public channel is commonly utilized in the majority of related existing works (e.g., [9,10,11,12,13,14,15,16]). It is important to note that all nodes, including Eve, have access to all the information transmitted over the public channel.

**Figure 2:**
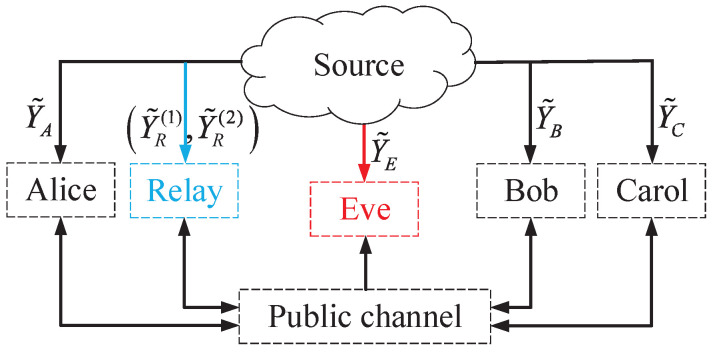
Group SK or PK generation in the DMS model.

Based on the above star network and model assumptions, the DMS model for group SK/PK generation is shown in Figure 2. The observations of Alice, Bob, and Carlo on random source are ${\tilde{Y}}_{A}$, ${\tilde{Y}}_{B}$, and ${\tilde{Y}}_{C}$, respectively. Moreover, the observations of the relay and Eve on random sources are $\left({\tilde{Y}}_{R}^{\left(1\right)},{\tilde{Y}}_{R}^{\left(2\right)}\right)$ and ${\tilde{Y}}_{E}$, respectively. From their sequence of observations on random sources and information transmitted over the public channel, the legitimate members can generate an SK and a PK shared within the group. The SK is required to be confidential from Eve, whereas both the relay and Eve have no knowledge of the PK. The maximum achievable SK/PK rate is termed as its capacity. Thus, any achievable SK/PK rate naturally serves as a lower bound for its capacity.

## 3. Group Secret and Private Key Generation Scheme

$G_{1}$ is considered to be the reference channel, and the relay enables all the legitimate members to obtain the observations of the reference channel. The proposed CJ-based group SK and PK generation scheme consists of two stages: channel measurement and key agreement.

### 3.1. Channel Measurement

Channel measurements are repeatedly collected by the group members, based on the sampling of the legitimate communication channels $G_{1}$, $G_{2}$, and $G_{3}$. Each fading block is divided into three time slots with $T_{1}$, $T_{2}$, and $T_{3}$ symbol times, where $T_{1}+T_{2}+T_{3}=T$. Additionally, assume that all transmitters employ a uniform transmit power of *P* in the training phase, and each receiver experiences a noise power of $\sigma^{2}$. The details of the training phase for channel measurements are listed as follows.

- In the first time slot, a predetermined sequence $S_{1}={\left[\underset{T_{1}}{\underset{︸}{\sqrt{P},\ldots ,\sqrt{P}}}\right]}^{\prime }$ is transmitted by the relay; thus, Alice, Bob, and Carlo receive $T_{1}$-sample sequence:
  (1) $$\begin{array}{c} Y_{A}^{\left(1\right)}=G_{1}S_{1}+N_{A}^{\left(1\right)}, \end{array}$$
  (2) $$\begin{array}{c} Y_{B}^{\left(1\right)}=G_{2}S_{1}+N_{B}^{\left(1\right)}, \end{array}$$
  (3) $$\begin{array}{c} Y_{C}^{\left(1\right)}=G_{3}S_{1}+N_{C}^{\left(1\right)}, \end{array}$$
  where $N_{A}^{\left(1\right)}$, $N_{B}^{\left(1\right)}$, and $N_{C}^{\left(1\right)}$ are Gaussian noise vectors of size $T_{1}\times 1$. Then, Alice, Bob, and Carlo obtain the estimates of channels $G_{1}$, $G_{2}$, and $G_{3}$, which are given by
  (4) $$\begin{array}{cc} {\tilde{Y}}_{A} & =G_{1}+N_{A}, \end{array}$$
  (5) $$\begin{array}{cc} {\tilde{Y}}_{B} & =G_{2}+N_{B}, \end{array}$$
  (6) $$\begin{array}{cc} {\tilde{Y}}_{C} & =G_{3}+N_{C}, \end{array}$$
  respectively, with $N_{A},N_{B},N_{C}∼\mathrm{CN}\left(0,\sigma_{1}^{2}\right)$ and $\sigma_{1}^{2}≜\frac{\sigma^{2}}{T_{1}P}$.
- In the second time slot, based on the concept of CJ, Alice and Bob send a known sequence $S_{2}={\left[\underset{T_{2}}{\underset{︸}{\sqrt{P},\ldots ,\sqrt{P}}}\right]}^{\prime }$ at the same time; thus, the relay receives
  (7) $$\begin{array}{cc} Y_{R}^{\left(2\right)} & =G_{1}S_{2}+G_{2}S_{2}+N_{R}^{\left(2\right)}. \end{array}$$
  Then, the relay obtains the superimposed channel estimates regarding $G_{1}$ and $G_{2}$:
  (8) $$\begin{array}{cc} {\tilde{Y}}_{R}^{\left(1\right)} & =G_{1}+G_{2}+N_{R}^{\left(1\right)}, \end{array}$$
  where $N_{R}^{\left(1\right)}∼\mathrm{CN}\left(0,\sigma_{2}^{2}\right)$ and $\sigma_{2}^{2}≜\frac{\sigma^{2}}{T_{2}P}$.
- In the third time slot, based on the concept of CJ, Alice and Carlo simultaneously transmit a known sequence $S_{3}={\left[\underset{T_{3}}{\underset{︸}{\sqrt{P},\ldots ,\sqrt{P}}}\right]}^{\prime }$; thus, the relay receives
  (9) $$\begin{array}{cc} Y_{R}^{\left(3\right)} & =G_{1}S_{3}+G_{3}S_{3}+N_{R}^{\left(3\right)}. \end{array}$$
  Then, the relay obtains the superimposed channel estimates regarding $G_{1}$ and $G_{3}$:
  (10) $$\begin{array}{cc} {\tilde{Y}}_{R}^{\left(2\right)} & =G_{1}+G_{3}+N_{R}^{\left(2\right)}, \end{array}$$
  where $N_{R}^{\left(2\right)}∼\mathrm{CN}\left(0,\sigma_{3}^{2}\right)$ and $\sigma_{3}^{2}≜\frac{\sigma^{2}}{T_{3}P}$.

Note that during each fading block, each member acquires one random channel measurement. Consequently, for every *n* fading blocks, Alice, Bob, and Carlo obtain independent and identically distributed (i.i.d.) *n*-dimensional vectors ${\tilde{Y}}_{A}$, ${\tilde{Y}}_{B}$, and ${\tilde{Y}}_{C}$, respectively. Since the relay can obtain channel measurements in both $T_{2}$ and $T_{3}$ time slots, the relay obtains *n*-dimensional sequences ${\tilde{Y}}_{R}^{\left(1\right)}$ and ${\tilde{Y}}_{R}^{\left(2\right)}$.

### 3.2. Key Agreement

After the channel measurement stage, there is still no common randomness among the group members. Slepian–Wolf coding [25] is further employed in the key agreement stage, enabling group members to generate SKs and PKs. The key agreement is based on a two-step public discussion:

1. The first step: The first step involves the relay utilizing Slepian–Wolf coding to transmit specific bits of helper data derived from its observations over the public channel. This process allows Bob and Carlo to independently recover the relay’s observations ${\tilde{Y}}_{R}^{\left(1\right)}$ and ${\tilde{Y}}_{R}^{\left(2\right)}$, respectively, and subsequently obtain estimates of the reference channel $G_{1}$.
2. The second step: The second step involves Alice employing Slepian–Wolf coding to send particular bits of helper data based on her observations through the public channel. This allows Bob and Carlo to recover Alice’s observation ${\tilde{Y}}_{A}$ by leveraging the correlated estimates from the reference channel $G_{1}$. Subsequently, the legitimate members establish a group SK or PK.

In the following subsections, the details of the aforementioned two public discussion steps are provided.

#### 3.2.1. The First Step of Public Discussion

The i.i.d. *n*-dimensional sequences ${\tilde{Y}}_{R}^{\left(1\right)}$ and ${\tilde{Y}}_{R}^{\left(2\right)}$ at the relay are transformed into quantized vectors ${\tilde{Y}}_{R}^{\left(1\right),\Delta }={\left[{\tilde{Y}}_{R}^{\left(1\right),\Delta }\left(1\right),\ldots ,{\tilde{Y}}_{R}^{\left(1\right),\Delta }\left(n\right)\right]}^{\prime }$ and ${\tilde{Y}}_{R}^{\left(2\right),\Delta }={\left[{\tilde{Y}}_{R}^{\left(2\right),\Delta }\left(1\right),\ldots ,{\tilde{Y}}_{R}^{\left(2\right),\Delta }\left(n\right)\right]}^{\prime }$, where ${\tilde{Y}}_{R}^{\left(1\right),\Delta }\left(i\right)$ and ${\tilde{Y}}_{R}^{\left(2\right),\Delta }\left(i\right)$ are quantized versions of ${\tilde{Y}}_{R}^{\left(1\right)}\left(i\right)$ and ${\tilde{Y}}_{R}^{\left(2\right)}\left(i\right)$ with quantization interval Δ, respectively. Similarly, Bob and Carlo transform ${\tilde{Y}}_{B}$ and ${\tilde{Y}}_{C}$ into ${\tilde{Y}}_{B}^{\Delta }={\left[{\tilde{Y}}_{B}^{\Delta }\left(1\right),\ldots ,{\tilde{Y}}_{B}^{\Delta }\left(n\right)\right]}^{\prime }$ and ${\tilde{Y}}_{C}^{\Delta }={\left[{\tilde{Y}}_{C}^{\Delta }\left(1\right),\ldots ,{\tilde{Y}}_{C}^{\Delta }\left(n\right)\right]}^{\prime }$, respectively. The relay randomly divides the typical ${\tilde{Y}}_{R}^{\left(1\right),\Delta }$ and ${\tilde{Y}}_{R}^{\left(2\right),\Delta }$ sequences into non-overlapping bins and then sends the bin number as helper data to Bob and Carlo. Based on Slepian-Wolf coding, the relay needs to, respectively, send $H\left({\tilde{Y}}_{R}^{\left(1\right),\Delta }\left|{\tilde{Y}}_{B}^{\Delta }\right.\right)$ and $H\left({\tilde{Y}}_{R}^{\left(2\right),\Delta }\left|{\tilde{Y}}_{C}^{\Delta }\right.\right)$ bits of information through the public channel to Bob and Carlo, where $H\left(X\left|Y\right.\right)$ represents the conditional entropy of a random variable *X* given *Y*. By combining the bin number transmitted over the public channel with their own observations, Bob and Carlo can recover ${\tilde{Y}}_{R}^{\left(1\right)}$ and ${\tilde{Y}}_{R}^{\left(2\right)}$ with the probability being arbitrarily close to 1. After that, combining ${\tilde{Y}}_{B}$ and ${\tilde{Y}}_{R}^{\left(1\right)}$, Bob can obtain an estimation sequence of $G_{1}$, denoted by ${\hat{G}}_{1,B}$. Combining ${\tilde{Y}}_{C}$ and ${\tilde{Y}}_{R}^{\left(2\right)}$, Carlo can obtain an estimation sequence of $G_{1}$, denoted by ${\hat{G}}_{1,C}$.

#### 3.2.2. The Second Step of Public Discussion

After the first step, Alice, Bob, and Carlo all obtain the correlated estimates of $G_{1}$, thereby enhancing common randomness. However, due to the presence of noise, further agreement is still required. Alice transforms ${\tilde{Y}}_{A}$ into ${\tilde{Y}}_{A}^{\Delta }={\left[{\tilde{Y}}_{A}^{\Delta }\left(1\right),\ldots ,{\tilde{Y}}_{A}^{\Delta }\left(n\right)\right]}^{\prime }$. Similarly, Bob and Carlo transform ${\hat{G}}_{1,B}$ and ${\hat{G}}_{1,C}$ into ${\hat{G}}_{1,B}^{\Delta }={\left[{\hat{G}}_{1,B}^{\Delta }\left(1\right),\ldots ,{\hat{G}}_{1,B}^{\Delta }\left(n\right)\right]}^{\prime }$ and ${\hat{G}}_{1,C}^{\Delta }={\left[{\hat{G}}_{1,C}^{\Delta }\left(1\right),\ldots ,{\hat{G}}_{1,C}^{\Delta }\left(n\right)\right]}^{\prime }$, respectively. To generate an SK and a PK with different security permission levels, Alice needs to adopt two different sub-bin partitioning methods according to Slepian–Wolf coding.

- Alice randomly and independently partitions the typical ${\tilde{Y}}_{A}^{\Delta }$ sequences into $2^{nR_{31}}$ bins, with each bin having $2^{nR_{32}^{SK}}$ sub-bins, where
  (11) $$\begin{array}{cc} R_{31} & =\max\left\{H\left({\tilde{Y}}_{A}^{\Delta }\left|{\hat{G}}_{1,B}^{\Delta }\right.\right),H\left({\tilde{Y}}_{A}^{\Delta }\left|{\hat{G}}_{1,C}^{\Delta }\right.\right)\right\}, \end{array}$$
  (12) $$\begin{array}{cc} R_{32}^{SK} & ={\left[H\left({\tilde{Y}}_{A}^{\Delta }\right)-\max\left\{H\left({\tilde{Y}}_{A}^{\Delta }\left|{\hat{G}}_{1,B}^{\Delta }\right.\right),H\left({\tilde{Y}}_{A}^{\Delta }\left|{\hat{G}}_{1,C}^{\Delta }\right.\right)\right\}\right]}^{+}. \end{array}$$
- Alice randomly and independently partitions the typical ${\tilde{Y}}_{A}^{\Delta }$ sequences into $2^{nR_{31}}$ bins, with each bin having $2^{nR_{32}^{PK}}$ sub-bins, where
  (13) $$\begin{array}{c} R_{32}^{PK}={\left[H\left({\tilde{Y}}_{A}^{\Delta }\left|{\tilde{Y}}_{R}^{\left(1\right),\Delta },{\tilde{Y}}_{R}^{\left(2\right),\Delta }\right.\right)-\max\left\{H\left({\tilde{Y}}_{A}^{\Delta }\left|{\hat{G}}_{1,B}^{\Delta }\right.\right),H\left({\tilde{Y}}_{A}^{\Delta }\left|{\hat{G}}_{1,C}^{\Delta }\right.\right)\right\}\right]}^{+}. \end{array}$$

Hence, each sequence has two indices: bin number and index within the bin. Alice takes the index within the bin of two different partitioning methods as an SK and a PK, respectively, and then sends the bin number as the helper data to Bob and Carlo. Alice needs to send $\max\left\{H\left({\tilde{Y}}_{A}^{\Delta }\left|{\hat{G}}_{1,B}^{\Delta }\right.\right),H\left({\tilde{Y}}_{A}^{\Delta }\left|{\hat{G}}_{1,C}^{\Delta }\right.\right)\right\}$ bits of information through the public channel. By combining their own observations ${\hat{G}}_{1,B}$, ${\hat{G}}_{1,C}$ and helper data, Bob and Carlo can recover ${\tilde{Y}}_{A}$ with the probability being arbitrarily close to 1. Thus, Bob and Carlo can both recover the SK and PK.

**Remark 1.**  *From the channel measurement and key agreement stages, it is evident that the estimates of the reference channel $G_{1}$ play a crucial role in enabling all legitimate nodes to share a group key. During the channel measurement stage, Alice obtains an estimation of $G_{1}$, as shown in (4). Bob, on the other hand, only obtains an estimation of $G_{2}$, i.e., ${\tilde{Y}}_{B}$, as shown in (5). However, following the first step of public discussion in the key agreement stage, Bob gains access to the relay’s observation ${\tilde{Y}}_{R}^{\left(1\right)}$. As illustrated in (8), ${\tilde{Y}}_{R}^{\left(1\right)}$ encompasses the term $G_{1}+G_{2}$, allowing Bob to employ a ZF method to estimate $G_{1}$ based on ${\tilde{Y}}_{B}$ and ${\tilde{Y}}_{R}^{\left(1\right)}$. Similarly, Carlo can also obtain an estimation of $G_{1}$. Subsequently, during the second step of public discussion, all legitimate nodes can leverage the correlated estimates of $G_{1}$ to generate a common randomness as well as a group key.*

**Remark 2.**  *On the other hand, both the relay and Eve can only acquire superimposed channel observations from both the reference channel $G_{1}$ and another channel, either $G_{2}$ or $G_{3}$. Consequently, they are unable to completely eliminate the influences of channels $G_{2}$ and $G_{3}$ when estimating $G_{1}$. As a result, the security of the reference channel-based group key generation can be significantly enhanced.*

**Remark 3.**  *Note that the group SK and PK generation scheme can be directly extended to scenarios involving N(N>3) legitimate nodes, where legitimate members $U_{1},U_{2},\ldots ,U_{N}$ and the relay collectively form a star network, and the channel gain between $U_{i}(i=1,2,\ldots ,N$) and the relay is denoted as $G_{i}$. In this case, the coherence time T is divided into N time slots, i.e., $T_{1},T_{2},\ldots ,T_{N}$. In the channel measurement stage, the legitimate members $U_{1},U_{2},\ldots ,U_{N}$ obtain their respective observations of the relay channel $G_{1},G_{2},\ldots ,G_{N}$, while the relay acquires superimposed channel observations regarding $G_{1}$ and $G_{j}(j=2,3,\ldots ,N$). In the key agreement stage, $U_{2},U_{3},\ldots ,U_{N}$ obtain estimates of channel $G_{1}$ through the first key agreement. Then, they agree on the same SK and PK as $U_{1}$ through the second key agreement.*

## 4. Performance Analysis

This section is dedicated to assessing the upper and lower bounds on the SK/PK capacity. The lower bounds are derived based on the proposed group key generation scheme in the previous section, while the upper bounds are derived by the corresponding formulated enhanced DMS models.

### 4.1. SK/PK Lower Bound

Based on (11), (13), and the findings in [10], an achievable SK or PK rate is given by

(14) $$\begin{array}{cc} R_{s} & =\min\left\{I\left({\tilde{Y}}_{A};{\hat{G}}_{1,B}\right),I\left({\tilde{Y}}_{A};{\hat{G}}_{1,C}\right)\right\}, \end{array}$$

(15) $$\begin{array}{cc} R_{p} & ={\left[\min\left\{I\left({\tilde{Y}}_{A};{\hat{G}}_{1,B}\right),I\left({\tilde{Y}}_{A};{\hat{G}}_{1,C}\right)\right\}-I\left({\tilde{Y}}_{A};{\tilde{Y}}_{R}^{\left(1\right)},{\tilde{Y}}_{R}^{\left(2\right)}\right)\right]}^{+}, \end{array}$$

which represents a lower bound on the SK or PK capacity, respectively. Note that $I\left({\tilde{Y}}_{A};{\tilde{Y}}_{R}^{\left(1\right)},{\tilde{Y}}_{R}^{\left(2\right)}\right)$ denotes the information leaked to the relay.

#### 4.1.1. Derivation of $R_{s}$

Assuming that Bob and Carlo utilize the ZF method to obtain the observations of the reference channel $G_{1}$, ${\hat{G}}_{1,B}$ and ${\hat{G}}_{1,C}$ are given by

(16) $$\begin{array}{cc} {\hat{G}}_{1,B} & ={\tilde{Y}}_{R}^{\left(1\right)}-{\tilde{Y}}_{B}=G_{1}+N_{R}^{\left(1\right)}-N_{B}, \end{array}$$

(17) $$\begin{array}{cc} {\hat{G}}_{1,C} & ={\tilde{Y}}_{R}^{\left(2\right)}-{\tilde{Y}}_{C}=G_{1}+N_{R}^{\left(2\right)}-N_{C}, \end{array}$$

respectively. The calculation of the correlation coefficient between ${\tilde{Y}}_{A}$ and ${\hat{G}}_{1,B}$ is given by

(18) $$\begin{array}{c} \begin{array}{cc} \rho_{{\tilde{Y}}_{A},{\hat{G}}_{1,B}}^{2} & =\frac{{\mathrm{Cov}}^{2}\left({\tilde{Y}}_{A},{\hat{G}}_{1,B}\right)}{\mathrm{Var}\left({\tilde{Y}}_{A}\right)\mathrm{Var}\left({\hat{G}}_{1,B}\right)}\\ & =\frac{p_{A}^{2}}{\left(p_{A}+\sigma_{1}^{2}\right)\left(p_{A}+\sigma_{1}^{2}+\sigma_{2}^{2}\right)}. \end{array} \end{array}$$

Thus, $I\left({\tilde{Y}}_{A};{\hat{G}}_{1,B}\right)$ is given by

(19) $$\begin{array}{c} \begin{array}{cc} I\left({\tilde{Y}}_{A};{\hat{G}}_{1,B}\right) & =-\log_{2}\left(1-\rho_{{\tilde{Y}}_{A},{\hat{G}}_{1,B}}^{2}\right)\\ \; & =\log_{2}\left(1+\frac{p_{A}^{2}}{p_{A}\left(2\sigma_{1}^{2}+\sigma_{2}^{2}\right)+\sigma_{1}^{4}+\sigma_{1}^{2}\sigma_{2}^{2}}\right). \end{array} \end{array}$$

Similarly, $I\left({\tilde{Y}}_{A};{\hat{G}}_{1,C}\right)$ is given by

(20) $$\begin{array}{c} I\left({\tilde{Y}}_{A};{\hat{G}}_{1,C}\right)=\log_{2}\left(1+\frac{p_{A}^{2}}{p_{A}\left(2\sigma_{1}^{2}+\sigma_{3}^{2}\right)+\sigma_{1}^{4}+\sigma_{1}^{2}\sigma_{3}^{2}}\right) \end{array}$$

Hence, the expression of $R_{s}$ can be obtained by substituting (19) and (20) into (14).

#### 4.1.2. Derivation of $R_{p}$

According to (14) and (15), one can observe that $R_{s}$ and $R_{p}$ only differ by one term. Therefore, only the expression of $I\left({\tilde{Y}}_{A};{\tilde{Y}}_{R}^{\left(1\right)},{\tilde{Y}}_{R}^{\left(2\right)}\right)$ needs to be derived, which is calculated as

(21) $$\begin{array}{c} \begin{array}{cc} I\left({\tilde{Y}}_{A};{\tilde{Y}}_{R}^{\left(1\right)},{\tilde{Y}}_{R}^{\left(2\right)}\right) & =h\left({\tilde{Y}}_{A}\right)+h\left({\tilde{Y}}_{R}^{\left(1\right)},{\tilde{Y}}_{R}^{\left(2\right)}\right)-h\left({\tilde{Y}}_{A},{\tilde{Y}}_{R}^{\left(1\right)},{\tilde{Y}}_{R}^{\left(2\right)}\right)\\ \; & =\log_{2}\left(\frac{\left(p_{A}+\sigma_{1}^{2}\right)\left|C_{{\tilde{Y}}_{R}^{\left(1\right)}{\tilde{Y}}_{R}^{\left(2\right)}}\right|}{\left|C_{{\tilde{Y}}_{A}{\tilde{Y}}_{R}^{\left(1\right)}{\tilde{Y}}_{R}^{\left(2\right)}}\right|}\right), \end{array} \end{array}$$

where the covariance matrices $C_{{\tilde{Y}}_{R}^{\left(1\right)}{\tilde{Y}}_{R}^{\left(2\right)}}$ and $C_{{\tilde{Y}}_{A}{\tilde{Y}}_{R}^{\left(1\right)}{\tilde{Y}}_{R}^{\left(2\right)}}$ can be given by

(22) $$C_{{\tilde{Y}}_{R}^{\left(1\right)}{\tilde{Y}}_{R}^{\left(2\right)}}=\left[\begin{array}{cc} p_{A}+p_{B}+\sigma_{2}^{2} & p_{A}\\ p_{A} & p_{A}+p_{C}+\sigma_{3}^{2} \end{array}\right],$$

(23) $$C_{{\tilde{Y}}_{A}{\tilde{Y}}_{R}^{\left(1\right)}{\tilde{Y}}_{R}^{\left(2\right)}}=\left[\begin{array}{ccc} p_{A}+\sigma_{1}^{2} & p_{A} & p_{A}\\ p_{A} & p_{A}+p_{B}+\sigma_{2}^{2} & p_{A}\\ p_{A} & p_{A} & p_{A}+p_{C}+\sigma_{3}^{2} \end{array}\right],$$

respectively. Hence, the expression of $R_{p}$ can be obtained by substituting (19), (20), and (21) into (15).

### 4.2. The SK/PK Upper Bound

**Figure 3:**
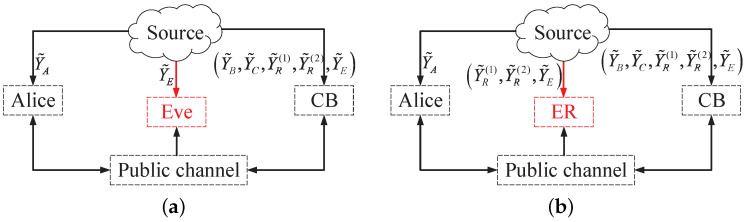
Group SK and PK generation in the enhanced DMS models. (**a**) SK. (**b**) PK.

To derive the SK/PK upper bound, we streamline the DMS model depicted in Figure 2. Assume that Bob and Carlo are colluding and can exchange all of their respective observed information. Thus, Bob and Carlo can be considered to be one node, defined as node CB. The enhanced DMS model with respect to group SK/PK generation is shown in Figure 3.

In the formulated enhanced DMS model for SK generation, the observations of the relay and Eve are known by either Alice or CB. First, we consider the scenario where CB has access to both the observations of the relay and Eve, as illustrated in Figure 3a. As a result, the DMS model presented in Figure 3 reduces to a standard point-to-point DMS model. In this model, Alice, CB, and Eve, respectively, have access to ${\tilde{Y}}_{A}$, the combined set of $\left({\tilde{Y}}_{B},{\tilde{Y}}_{C},{\tilde{Y}}_{R}^{\left(1\right)},{\tilde{Y}}_{R}^{\left(2\right)},{\tilde{Y}}_{E}\right)$ and ${\tilde{Y}}_{E}$. According to [26], the SK capacity is upper bounded by $I\left({\tilde{Y}}_{A};{\tilde{Y}}_{B},{\tilde{Y}}_{C},{\tilde{Y}}_{R}^{\left(1\right)},{\tilde{Y}}_{R}^{\left(2\right)},{\tilde{Y}}_{E}\left|{\tilde{Y}}_{E}\right.\right)=I\left({\tilde{Y}}_{A};{\tilde{Y}}_{B},{\tilde{Y}}_{C},{\tilde{Y}}_{R}^{\left(1\right)},{\tilde{Y}}_{R}^{\left(2\right)}\right)$. Second, assuming that the observations of the relay and Eve are known by Alice, the SK capacity is upper bounded by $I\left({\tilde{Y}}_{A},{\tilde{Y}}_{R}^{\left(1\right)},{\tilde{Y}}_{R}^{\left(2\right)},{\tilde{Y}}_{E};{\tilde{Y}}_{B},{\tilde{Y}}_{C}\left|{\tilde{Y}}_{E}\right.\right)=I\left({\tilde{Y}}_{A},{\tilde{Y}}_{R}^{\left(1\right)},{\tilde{Y}}_{R}^{\left(2\right)};{\tilde{Y}}_{B},{\tilde{Y}}_{C}\right)$.

For the enhanced DMS model for PK generation, assume that the relay and Eve are colluding, and thus the relay and Eve can be considered to be one node, defined as node ER. Additionally, assuming CB has access to the observations of the relay and Eve, as depicted in Figure 3b, the subsequent observations made by Alice, CB, and ER are ${\tilde{Y}}_{A}$, $\left({\tilde{Y}}_{B},{\tilde{Y}}_{C},{\tilde{Y}}_{R}^{\left(1\right)},{\tilde{Y}}_{R}^{\left(2\right)},{\tilde{Y}}_{E}\right)$ and $\left({\tilde{Y}}_{R}^{\left(1\right)},{\tilde{Y}}_{R}^{\left(2\right)},{\tilde{Y}}_{E}\right)$, respectively. Therefore, according to [26], the upper bound on PK capacity is $I\left({\tilde{Y}}_{A};{\tilde{Y}}_{B},{\tilde{Y}}_{C},{\tilde{Y}}_{R}^{\left(1\right)},{\tilde{Y}}_{R}^{\left(2\right)},{\tilde{Y}}_{E}\left|{\tilde{Y}}_{R}^{\left(1\right)},{\tilde{Y}}_{R}^{\left(2\right)},{\tilde{Y}}_{E}\right.\right)=I\left({\tilde{Y}}_{A};{\tilde{Y}}_{B},{\tilde{Y}}_{C}\left|{\tilde{Y}}_{R}^{\left(1\right)},{\tilde{Y}}_{R}^{\left(2\right)}\right.\right)$.

According to the above analysis, the upper bounds on SK and PK capacities can be given by

(24) $$\begin{array}{cc} C_{s}^{U} & =\min\left\{I\left({\tilde{Y}}_{A};{\tilde{Y}}_{B},{\tilde{Y}}_{C},{\tilde{Y}}_{R}^{\left(1\right)},{\tilde{Y}}_{R}^{\left(2\right)}\right),I\left({\tilde{Y}}_{A},{\tilde{Y}}_{R}^{\left(1\right)},{\tilde{Y}}_{R}^{\left(2\right)};{\tilde{Y}}_{B},{\tilde{Y}}_{C}\right)\right\} \end{array}$$

(25) $$\begin{array}{cc} C_{p}^{U} & =I\left({\tilde{Y}}_{A};{\tilde{Y}}_{B},{\tilde{Y}}_{C}\left|{\tilde{Y}}_{R}^{\left(1\right)},{\tilde{Y}}_{R}^{\left(2\right)}\right.\right) \end{array}$$

#### 4.2.1. Upper Bound on SK Capacity

For (24), its first term can be calculated as

(26) $$\begin{array}{c} \begin{array}{cc} \; & \;I\left({\tilde{Y}}_{A};{\tilde{Y}}_{B},{\tilde{Y}}_{C},{\tilde{Y}}_{R}^{\left(1\right)},{\tilde{Y}}_{R}^{\left(2\right)}\right)\\ & =h\left({\tilde{Y}}_{A}\right)+h\left({\tilde{Y}}_{B},{\tilde{Y}}_{C},{\tilde{Y}}_{R}^{\left(1\right)},{\tilde{Y}}_{R}^{\left(2\right)}\right)-h\left({\tilde{Y}}_{A};{\tilde{Y}}_{B},{\tilde{Y}}_{C},{\tilde{Y}}_{R}^{\left(1\right)},{\tilde{Y}}_{R}^{\left(2\right)}\right)\\ \; & =\log_{2}\frac{\left(p_{A}+\sigma_{1}^{2}\right)\left|C_{{\tilde{Y}}_{B}{\tilde{Y}}_{C}{\tilde{Y}}_{R}^{\left(1\right)}{\tilde{Y}}_{R}^{\left(2\right)}}\right|}{\left|C_{{\tilde{Y}}_{A}{\tilde{Y}}_{B}{\tilde{Y}}_{C}{\tilde{Y}}_{R}^{\left(1\right)}{\tilde{Y}}_{R}^{\left(2\right)}}\right|}, \end{array} \end{array}$$

where the covariance matrices $C_{{\tilde{Y}}_{B}{\tilde{Y}}_{C}{\tilde{Y}}_{R}^{\left(1\right)}{\tilde{Y}}_{R}^{\left(2\right)}}$ and $C_{{\tilde{Y}}_{A}{\tilde{Y}}_{B}{\tilde{Y}}_{C}{\tilde{Y}}_{R}^{\left(1\right)}{\tilde{Y}}_{R}^{\left(2\right)}}$ can be given by

(27) $$C_{{\tilde{Y}}_{B}{\tilde{Y}}_{C}{\tilde{Y}}_{R}^{\left(1\right)}{\tilde{Y}}_{R}^{\left(2\right)}}=\left[\begin{array}{cccc} p_{B}+\sigma_{1}^{2} & 0 & p_{B} & 0\\ 0 & p_{C}+\sigma_{1}^{2} & 0 & p_{C}\\ p_{B} & 0 & p_{A}+p_{B}+\sigma_{2}^{2} & p_{A}\\ 0 & p_{C} & p_{A} & p_{A}+p_{C}+\sigma_{3}^{2} \end{array}\right],$$

(28) $$C_{{\tilde{Y}}_{A}{\tilde{Y}}_{B}{\tilde{Y}}_{C}{\tilde{Y}}_{R}^{\left(1\right)}{\tilde{Y}}_{R}^{\left(2\right)}}=\left[\begin{array}{ccccc} p_{A}\;+\;\sigma_{1}^{2} & 0 & 0 & p_{A} & p_{A}\\ 0 & p_{B}\;+\;\sigma_{1}^{2} & 0 & p_{B} & 0\\ 0 & 0 & p_{C}\;+\;\sigma_{1}^{2} & 0 & p_{C}\\ p_{A} & p_{B} & 0 & p_{A}\;+\;p_{B}\;+\;\sigma_{2}^{2} & p_{A}\\ p_{A} & 0 & p_{C} & p_{A} & p_{A}\;+\;p_{C}\;+\;\sigma_{3}^{2} \end{array}\right],$$

respectively.

Likewise, the computation of the second term in (24) is given by

(29) $$\begin{array}{c} \begin{array}{cc} I\left({\tilde{Y}}_{A},{\tilde{Y}}_{R}^{\left(1\right)},{\tilde{Y}}_{R}^{\left(2\right)};{\tilde{Y}}_{B},{\tilde{Y}}_{C}\right)\; & =h\left({\tilde{Y}}_{A},{\tilde{Y}}_{R}^{\left(1\right)},{\tilde{Y}}_{R}^{\left(2\right)}\right)+h\left({\tilde{Y}}_{B},{\tilde{Y}}_{C}\right)-h\left({\tilde{Y}}_{A},{\tilde{Y}}_{B},{\tilde{Y}}_{C},{\tilde{Y}}_{R}^{\left(1\right)},{\tilde{Y}}_{R}^{\left(2\right)}\right)\\ \; & =\log_{2}\frac{\left|C_{{\tilde{Y}}_{A}{\tilde{Y}}_{R}^{\left(1\right)}{\tilde{Y}}_{R}^{\left(2\right)}}\right|\left|C_{{\tilde{Y}}_{B}{\tilde{Y}}_{C}}\right|}{\left|C_{{\tilde{Y}}_{A}{\tilde{Y}}_{B}{\tilde{Y}}_{C}{\tilde{Y}}_{R}^{\left(1\right)}{\tilde{Y}}_{R}^{\left(2\right)}}\right|}, \end{array} \end{array}$$

where $C_{{\tilde{Y}}_{A}{\tilde{Y}}_{R}^{\left(1\right)}{\tilde{Y}}_{R}^{\left(2\right)}}$ and $C_{{\tilde{Y}}_{A}{\tilde{Y}}_{B}{\tilde{Y}}_{C}{\tilde{Y}}_{R}^{\left(1\right)}{\tilde{Y}}_{R}^{\left(2\right)}}$ are given in (23) and (28), respectively, and $C_{{\tilde{Y}}_{B}{\tilde{Y}}_{C}}$ can be expressed as

(30) $$C_{{\tilde{Y}}_{B}{\tilde{Y}}_{C}}=\left[\begin{array}{cc} p_{B}+\sigma_{1}^{2} & 0\\ 0 & p_{C}+\sigma_{1}^{2} \end{array}\right].$$

#### 4.2.2. Upper Bound on PK Capacity

The expression of $C_{p}^{U}$ in (25) can be calculated as

(31) $$\begin{array}{c} \begin{array}{cc} \; & \;I\left({\tilde{Y}}_{A};{\tilde{Y}}_{B},{\tilde{Y}}_{C}\left|{\tilde{Y}}_{R}^{\left(1\right)},{\tilde{Y}}_{R}^{\left(2\right)}\right.\right)\\ \; & =I\left({\tilde{Y}}_{A};{\tilde{Y}}_{B},{\tilde{Y}}_{C},{\tilde{Y}}_{R}^{\left(1\right)},{\tilde{Y}}_{R}^{\left(2\right)}\right)-I\left({\tilde{Y}}_{A};{\tilde{Y}}_{R}^{\left(1\right)},{\tilde{Y}}_{R}^{\left(2\right)}\right)\\ \; & =h\left({\tilde{Y}}_{A},{\tilde{Y}}_{R}^{\left(1\right)},{\tilde{Y}}_{R}^{\left(2\right)}\right)+h\left({\tilde{Y}}_{B},{\tilde{Y}}_{C},{\tilde{Y}}_{R}^{\left(1\right)},{\tilde{Y}}_{R}^{\left(2\right)}\right)-h\left({\tilde{Y}}_{R}^{\left(1\right)},{\tilde{Y}}_{R}^{\left(2\right)}\right)-h\left({\tilde{Y}}_{A},{\tilde{Y}}_{B},{\tilde{Y}}_{C},{\tilde{Y}}_{R}^{\left(1\right)},{\tilde{Y}}_{R}^{\left(2\right)}\right)\\ \; & =\log_{2}\frac{\left|C_{{\tilde{Y}}_{A}{\tilde{Y}}_{R}^{\left(1\right)}{\tilde{Y}}_{R}^{\left(2\right)}}\right|\left|C_{{\tilde{Y}}_{B}{\tilde{Y}}_{C}{\tilde{Y}}_{R}^{\left(1\right)}{\tilde{Y}}_{R}^{\left(2\right)}}\right|}{\left|C_{{\tilde{Y}}_{R}^{\left(1\right)}{\tilde{Y}}_{R}^{\left(2\right)}}\right|\left|C_{{\tilde{Y}}_{A}{\tilde{Y}}_{B}{\tilde{Y}}_{C}{\tilde{Y}}_{R}^{\left(1\right)}{\tilde{Y}}_{R}^{\left(2\right)}}\right|}, \end{array} \end{array}$$

where $C_{{\tilde{Y}}_{A}{\tilde{Y}}_{R}^{\left(1\right)}{\tilde{Y}}_{R}^{\left(2\right)}}$, $C_{{\tilde{Y}}_{B}{\tilde{Y}}_{C}{\tilde{Y}}_{R}^{\left(1\right)}{\tilde{Y}}_{R}^{\left(2\right)}}$ and $C_{{\tilde{Y}}_{A}{\tilde{Y}}_{B}{\tilde{Y}}_{C}{\tilde{Y}}_{R}^{\left(1\right)}{\tilde{Y}}_{R}^{\left(2\right)}}$ are given in (23), (27), and (28), respectively, and $C_{{\tilde{Y}}_{R}^{\left(1\right)}{\tilde{Y}}_{R}^{\left(2\right)}}$ can be given by

(32) $$C_{{\tilde{Y}}_{R}^{\left(1\right)}{\tilde{Y}}_{R}^{\left(2\right)}}=\left[\begin{array}{cc} p_{A}+p_{B}+\sigma_{2}^{2} & p_{A}\\ p_{A} & p_{A}+p_{C}+\sigma_{3}^{2} \end{array}\right].$$

**Theorem 1.**  *In the high SNR scenario, when $T_{1}=T_{2}=T_{3}$, the upper and lower bounds maintain a fixed gap of $\log_{2}\left(\frac{3}{2}\right)$ BPCM for both the SK and PK.*

**Proof of Theorem 1.**  Please refer to Appendix A. □

## 5. Numerical Results

To verify the security performance of the proposed CJ-based key generation scheme, we numerically analyze the analytical results presented in previous sections. In this section, we consider a scenario with a group size of N(≥3). Especially, when N=3, $U_{1}$, $U_{2}$, and $U_{3}$ represent Alice, Bob, and Carlo, respectively. Moreover, the channel $G_{i}\left(i=1,2,\cdots ,N\right)$ is modeled as $G_{i}=g_{i}d_{G_{i}}^{-l/2}$, where $g_{i}∼CN\left(0,1\right)$, $d_{G_{i}}$ is the distance between the relay and $U_{i}$, and *l* is the pass-loss exponent. Therefore, $G_{i}∼CN\left(0,d_{G_{i}}^{l}\right)$. Additionally, to more accurately portray the influence of the parameter *T*, the metric unit utilized for measuring the key rate in this section is “bits per symbol time” (BPST). For brevity, the coherence time is divided into *N* time slots, each with a duration of $T_{0}=5$; all nodes have the same transmit power *P*; the distances between the group members $U_{i}\left(i=1,2,\cdots ,N\right)$ and the relay are assumed to be equal, denoted by *d* in meters, with a path loss coefficient of l=3. The distance value is within the range of 10 to 100 m, and the noise power is set as $\sigma^{2}=-50$ dBm [27].

**Figure 4:**
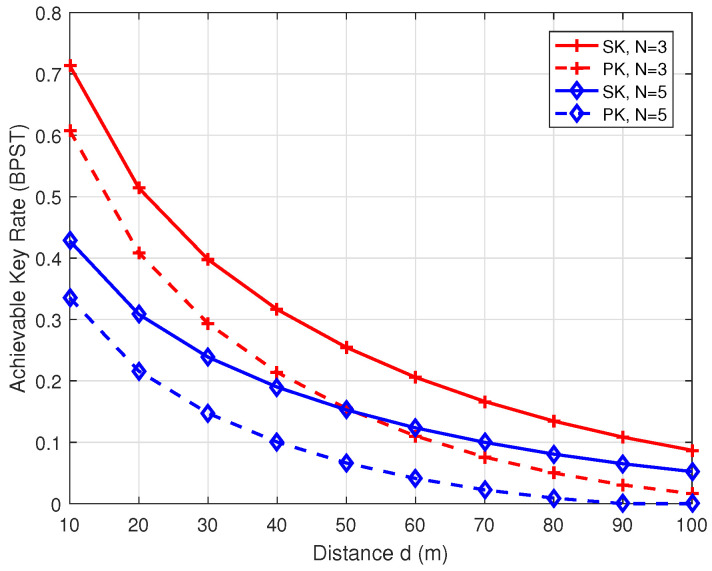
The achievable key rates of SK and PK versus the distance *d*.

Figure 4 shows the achievable SK/PK rate of the proposed group key generation scheme versus the distance *d*, with a transmit power of P=10 dBm and N=3 or 5. As depicted in the figure, both the SK and PK rates decrease as *d* increases, as longer distances result in lower received SNR at each receiver during the channel measurement process. For the scenario where N=3 and d=10 m, the achievable SK and PK rates are approximately 0.7 and 0.6 BPST, respectively. Furthermore, with N=5 and d=10 m, the SK and PK rates decrease to around 0.33 and 0.43 BPST, respectively. This indicates a difference of about 0.1 BPST in the achievable SK and PK rates for small values of *d*.

**Figure 5:**
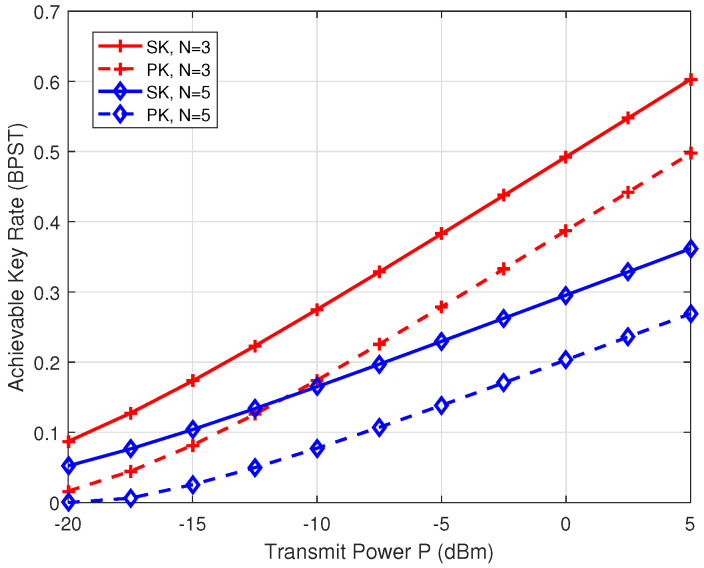
The achievable key rates of SK and PK versus the transmit power *P*.

Figure 5 showcases the SK and PK rates versus the transmit power *P*, with d=10 m and N=3 or 5. As shown in Figure 5, when N=3 and P=−10 dBm, the SK and PK rates are 0.275 BPST and 0.174 BPST, respectively. In addition, when N=3 and P=0 dBm, the SK and PK rates become 0.493 BPST and 0.387 BPST, respectively. One can observe that the achievable rates of the SK and PK increase as the transmit power *P* increases. This is because high transmit power improves the accuracy of channel measurement. Furthermore, when N=3 or 5, a noticeable difference of about 0.1 BPST exists between the SK and PK rates, illustrating the amount of information revealed to the relay, as described in (21).

**Figure 6:**
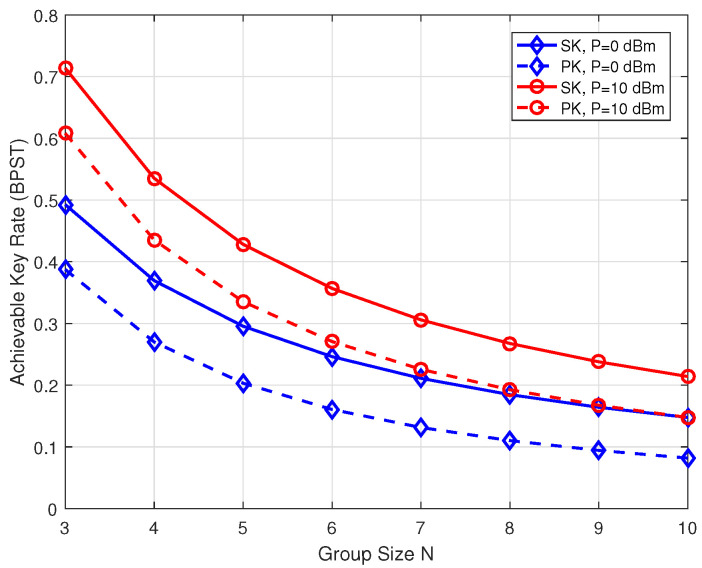
The achievable key rates of SK and PK as functions of group size *N*.

Figure 6 shows the impact of the group size on the achievable SK/PK rate, where d=10 m and P=0 or 10 dBm. As shown in this figure, when P=0 dBm, the SK and PK rates with N=3 are 0.493 BPST and 0.387 BPST, respectively. Furthermore, the achievable SK and PK rates with N=6 are 0.246 BPST and 0.161 BPST, respectively. One can observe that as the group size increases, both the SK and PK rates decrease. This decline can be attributed to the necessity for more time slots for channel measurement as the group size grows. Specifically, when N=3, the coherence time required is T=15. However, when N=6, the coherence time extends to T=30.

**Figure 7:**
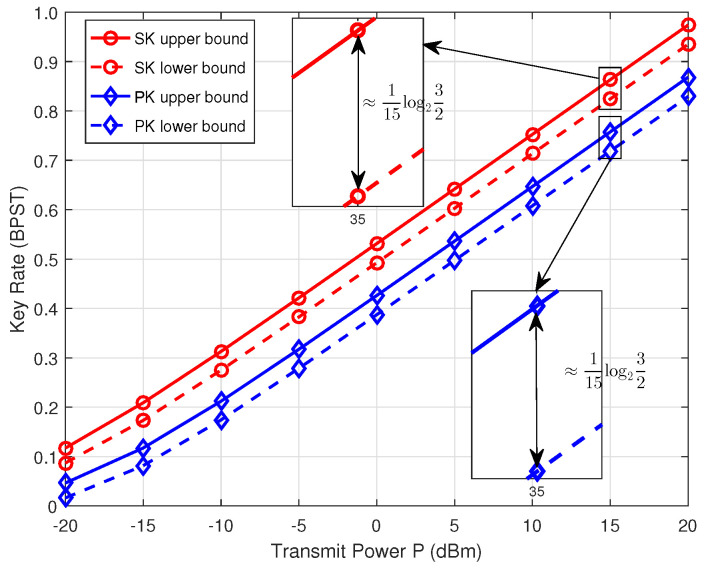
The SK/PK upper and lower bounds versus the transmit power *P*.

Figure 7 shows the SK/PK upper and lower bounds as they vary with the transmit power *P*, considering N=3 and d=10 m. The representation clearly shows that both these capacity bounds increase as the transmit power *P* increases. Furthermore, in scenarios characterized by high transmit power (i.e., high transmit SNR), a consistent gap of around $\frac{1}{15}\log_{2}\left(\frac{3}{2}\right)$ BPST is maintained between the SK/PK upper and lower bounds. This observation aligns with Theorem 1 through the transformation of the metric unit. One can observe that the key rates of SK and PK are notably large in the high SNR regime, which makes the gap negligible at high SNRs. Therefore, the proposed CJ-based physical layer group SK and PK generation scheme has a performance that is close to optimal bound.

**Figure 8:**
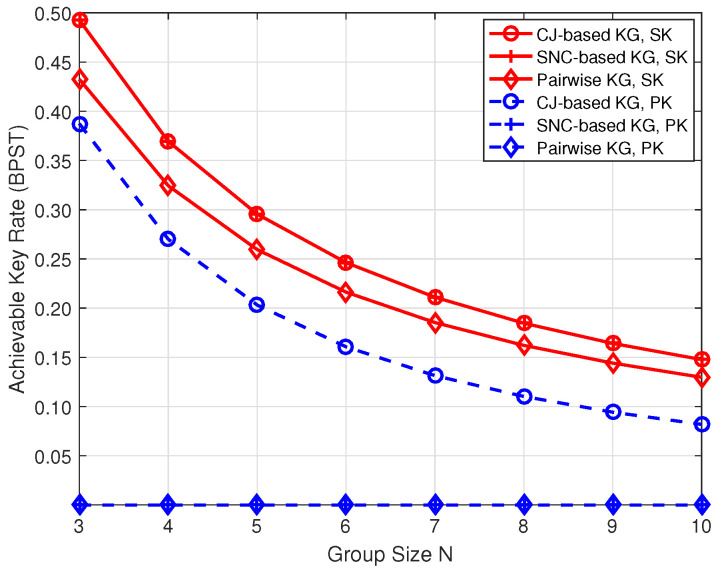
Performance comparison under three different key generation schemes.

Figure 8 compares the security performance among the proposed CJ-based, SNC-based [16] key generation (KG) schemes, and the traditional pairwise KG scheme [11,12,13,14,22], where d=100 m and P=30 dBm. As depicted in the figure, the SK rate of the CJ-based scheme equals that of the SNC-based scheme, both surpassing the pairwise scheme. Additionally, as the group size increases, the disparity in SK rates among the three schemes decreases gradually. This trend stems from the fact that when the group size is *N*, both the CJ-based and SNC-based schemes require a coherence time of ${NT}_{0}$, whereas the pairwise scheme necessitates ${(N+1)T}_{0}$. Consequently, the SK rates of the CJ-based and SNC-based schemes are $\frac{N+1}{N}$ times that of the pairwise scheme, with the rate gap narrowing as *N* increases. However, as the group size expands, the number of key agreements for the pairwise scheme also increases, leading to diminished key generation efficiency. Conversely, the key agreement times of the CJ-based and SNC-based schemes remain unaffected by group size expansion. Specifically, the CJ-based scheme requires two key agreements, while the SNC-based scheme requires only one key agreement. Moreover, the first key agreement stage of the CJ-based scheme plays a similar role to the secret sharing stage in the SNC-based scheme; thus, the key generation efficiency of the two schemes is similar. However, the distinction lies in the fact that the second key agreement stage of the CJ-based scheme can generate both an SK and a PK, while the key agreement of the SNC-based scheme solely generates SK. Similarly, the pairwise scheme can only generate the SK. Therefore, the PK rate of the SNC-based scheme and the pairwise scheme is always 0.

## 6. Conclusions

In this paper, we investigated the CJ-based group secret and private key generation scheme. The legitimate nodes within the group agree on an SK that is only kept secret from Eve, or a PK that is kept secret from both the relay and Eve. We constrained the SK and PK capacities by establishing their lower and upper bounds. The lower bounds were determined based on the ZF method, while the upper bounds were attained by analyzing the formulated enhanced DMS models. Moreover, we confirmed that these bounds closely align with each other, indicating their correlation. Particularly, in high SNR scenarios, a consistent difference of $\log_{2}\left(\frac{3}{2}\right)$ BPCM is evident between the SK/PK upper and lower bounds. Moreover, we also demonstrated that the proposed scheme significantly improves the security and efficiency of group key generation.

## Data Availability

The original contributions presented in the study are included in the article, further inquiries can be directed to the corresponding author.

## Appendix A. Proof of Theorem 1

When $T_{1}=T_{2}=T_{3}$, we can infer that $\sigma_{1}^{2}=\sigma_{2}^{2}=\sigma_{3}^{2}=3\sigma^{2}/PT$. For simplicity, we denote μ=Δ11σ12σ12=11σ22σ22=11σ32σ32. At high SNRs, $P/\delta^{2}$ and μ are sufficiently large. Moreover, we denote $\beta_{1}\overset{\Delta }{\;=}\left|C_{{\tilde{Y}}_{A}{\tilde{Y}}_{R}^{\left(1\right)}{\tilde{Y}}_{R}^{\left(2\right)}}\right|$, $\beta_{2}\overset{\Delta }{\;=}\left|C_{{\tilde{Y}}_{B}{\tilde{Y}}_{C}{\tilde{Y}}_{R}^{\left(1\right)}{\tilde{Y}}_{R}^{\left(2\right)}}\right|$ and $\beta_{3}\overset{\Delta }{\;=}\left|C_{{\tilde{Y}}_{A}{\tilde{Y}}_{B}{\tilde{Y}}_{C}{\tilde{Y}}_{R}^{\left(1\right)}{\tilde{Y}}_{R}^{\left(2\right)}}\right|$. Therefore, according to (23), (27), and (28), $\beta_{1}$, $\beta_{2}$ and $\beta_{3}$ can be calculated as

(A1) $$\begin{array}{cc} \beta_{1} & =p_{A}p_{B}p_{C}+\mu^{-1}\left(2p_{A}p_{B}+2p_{A}p_{C}+p_{B}p_{C}\right)+\mu^{-2}\left(3p_{A}+p_{B}+p_{C}\right)+\mu^{-3} \end{array}$$

(A2) $$\begin{array}{cc} \beta_{2} & =4\mu^{-1}p_{A}p_{B}p_{C}+\mu^{-2}\left(3p_{A}p_{B}+3p_{A}p_{C}+4p_{B}p_{C}\right)+2\mu^{-3}\left(p_{A}+p_{B}+p_{C}\right)+\mu^{-4} \end{array}$$

(A3) $$\begin{array}{cc} \beta_{3} & =8\mu^{-2}p_{A}p_{B}p_{C}\;+\;\mu^{-3}\left(5p_{A}p_{B}+5p_{A}p_{C}+4p_{B}p_{C}\right)+\mu^{-4}\left(3p_{A}+2p_{B}+2p_{C}\right)\;+\;\mu^{-5} \end{array}$$

### Appendix A.1. The SK Lower and Upper Bounds

To derive $R_{s}$, $I\left({\tilde{Y}}_{A};{\hat{G}}_{1,B}\right)$ in (19) is approximated as

(A4) $$\begin{array}{c} I\left({\tilde{Y}}_{A};{\hat{G}}_{1,B}\right)=\log_{2}\left(1+\frac{p_{A}^{2}}{3\mu^{-1}p_{A}+2\mu^{-2}}\right)\approx \log_{2}\frac{\mu p_{A}}{3}. \end{array}$$

Since $\sigma_{1}^{2}=\sigma_{2}^{2}=\sigma_{3}^{2}$, it can be observed that $I\left({\tilde{Y}}_{A};{\hat{G}}_{1,C}\right)=I\left({\tilde{Y}}_{A};{\hat{G}}_{1,B}\right)$ by comparing (19) and (20). Therefore, based on the expression of $R_{s}$ in (14), we can further infer that in the high SNR regime,

(A5) $$R_{s}\approx \log_{2}\frac{\mu p_{A}}{3}.$$

For the derivation of $C_{s}^{U}$, $I\left({\tilde{Y}}_{A};{\tilde{Y}}_{B},{\tilde{Y}}_{C},{\tilde{Y}}_{R}^{\left(1\right)},{\tilde{Y}}_{R}^{\left(2\right)}\right)$ in (26) is approximated as

(A6) $$I\left({\tilde{Y}}_{A};{\tilde{Y}}_{B},{\tilde{Y}}_{C},{\tilde{Y}}_{R}^{\left(1\right)},{\tilde{Y}}_{R}^{\left(2\right)}\right)=\log_{2}\frac{\left(p_{A}+\mu^{-1}\right)\beta_{2}}{\beta_{3}}\approx \log_{2}\frac{\mu p_{A}}{2}.$$

Similarly, $I\left({\tilde{Y}}_{A},{\tilde{Y}}_{R}^{\left(1\right)},{\tilde{Y}}_{R}^{\left(2\right)};{\tilde{Y}}_{B},{\tilde{Y}}_{C}\right)$ in (29) is approximated as

(A7) $$\begin{array}{cc} I\left({\tilde{Y}}_{A},{\tilde{Y}}_{R}^{\left(1\right)},{\tilde{Y}}_{R}^{\left(2\right)};{\tilde{Y}}_{B},{\tilde{Y}}_{C}\right) & =\log_{2}\frac{\beta_{1}\left[p_{B}p_{C}+\mu^{-1}\left(p_{B}+p_{C}\right)+\mu^{-2}\right]}{\beta_{3}}\\ \; & \approx \log_{2}\frac{\mu^{2}p_{B}p_{C}}{8}. \end{array}$$

Therefore, based on the expression of $C_{s}^{U}$ in (24), we can further infer that in the high SNR regime,

(A8) $$C_{s}^{U}\approx \log_{2}\frac{\mu p_{A}}{2}.$$

Consequently, combining (A5) and (A8), the difference between $C_{s}^{U}$ and $R_{s}$ is

(A9) $$\begin{array}{c} C_{s}^{U}-R_{s}\approx \log_{2}\frac{3}{2}. \end{array}$$

### Appendix A.2. The PK Lower and Upper Bounds

For the derivation of $R_{p}$, $I\left({\tilde{Y}}_{A};{\tilde{Y}}_{R}^{\left(1\right)},{\tilde{Y}}_{R}^{\left(2\right)}\right)$ in (21) is approximated as follows:

(A10) $$\begin{array}{c} I\left({\tilde{Y}}_{A};{\tilde{Y}}_{R}^{\left(1\right)},{\tilde{Y}}_{R}^{\left(2\right)}\right)\approx \log_{2}\left(1+\frac{p_{A}p_{B}+p_{A}p_{C}}{p_{B}p_{C}}\right). \end{array}$$

Therefore, the approximate expression of $R_{p}$ can be obtained by substituting (A4) and (A10) into (15):

(A11) $$R_{p}\approx \log_{2}\frac{\mu p_{A}p_{B}p_{C}}{3(p_{A}p_{B}+p_{A}p_{C}+p_{B}p_{C})}.$$

For the derivation of $C_{p}^{U}$, $I\left({\tilde{Y}}_{A};{\tilde{Y}}_{B},{\tilde{Y}}_{C}\left|{\tilde{Y}}_{R}^{\left(1\right)},{\tilde{Y}}_{R}^{\left(2\right)}\right.\right)$ in (31) is approximated as

(A12) $$\begin{array}{cc} I\left({\tilde{Y}}_{A};{\tilde{Y}}_{B},{\tilde{Y}}_{C}\left|{\tilde{Y}}_{R}^{\left(1\right)},{\tilde{Y}}_{R}^{\left(2\right)}\right.\right) & =\log_{2}\frac{\beta_{1}\beta_{2}}{\left[\left(p_{A}p_{B}\;+\;p_{A}p_{C}\;+\;p_{B}p_{C}\right)\;+\;\mu^{-1}\left(2p_{A}\;+\;p_{B}\;+\;p_{C}\right)\;+\;\mu^{-2}\right]\beta_{3}}\\ \; & \approx \log_{2}\frac{\mu p_{A}p_{B}p_{C}}{2(p_{A}p_{B}+p_{A}p_{C}+p_{B}p_{C})}. \end{array}$$

Therefore, based on the expression of $C_{p}^{U}$ in (25), we can further obtain that in the high SNR regime,

(A13) $$C_{p}^{U}\approx \log_{2}\frac{\mu p_{A}p_{B}p_{C}}{2(p_{A}p_{B}+p_{A}p_{C}+p_{B}p_{C})}.$$

Consequently, combining (A11) and (A13), the difference between $C_{p}^{U}$ and $R_{p}$ is

(A14) $$\begin{array}{c} C_{p}^{U}-R_{p}\approx \log_{2}\frac{3}{2}. \end{array}$$

## References

[1] Ahmad I., Shahabuddin S., Kumar T., Okwuibe J., Gurtov A., Ylianttila M. (2019). Security for 5G and beyond. IEEE Commun. Surv. Tuts..

[2] Nguyen V.-L., Lin P.-C., Cheng B.-C., Hwang R.-H., Lin Y.-D. (2021). Security and privacy for 6G: A survey on prospective technologies and challenges. IEEE Commun. Surv. Tuts..

[3] Porambage P., Gür G., Osorio D.P.M., Liyanage M., Gurtov A., Ylianttila M. (2021). The roadmap to 6G security and privacy. IEEE Open J. Commun. Soc..

[4] Halbouni A., Ong L.-Y., Leow M.-C. (2023). Wireless security protocols WPA3: A systematic literature review. IEEE Access..

[5] Du H., Wang J., Niyato D., Kang J., Xiong Z., Guizani M., Kim D.I. (2023). Rethinking wireless communication security in semantic Internet of Things. IEEE Wirel. Commun..

[6] Günlü O., Schaefer R.F. (2021). An optimality summary: Secret key agreement with physical unclonable functions. Entropy.

[7] Lin P.-H., Janda C.R., Jorswieck E.A., Schaefer R.F. (2020). Stealthy secret key generation. Entropy.

[8] Li G., Sun C., Zhang J., Jorswieck E., Xiao B., Hu A. (2019). Physical layer key generation in 5G and beyond wireless communications: Challenges and opportunities. Entropy.

[9] Truyen Thai C.D., Lee J., Quek T.Q.S. Secret group key generation in physical layer for mesh topology. Proceedings of the 2015 IEEE Global Communications Conference (GLOBECOM).

[10] Csiszar I., Narayan P. (2004). Secrecy capacities for multiple terminals. IEEE Trans. Inf. Theory.

[11] Ye C., Reznik A. Group secret key generation algorithms. Proceedings of the 2007 IEEE International Symposium on Information Theory (ISIT).

[12] Nitinawarat S., Ye C., Barg A., Narayan P., Reznik A. (2010). Secret key generation for a pairwise independent network model. IEEE Trans. Inf. Theory.

[13] Xu P., Cumanan K., Ding Z., Dai X., Leung K.K. (2016). Group secret key generation in wireless networks: Algorithms and rate optimization. IEEE Trans. Inf. Forensics Secur..

[14] Nitinawarat S., Narayan P. (2010). Perfect omniscience, perfect secrecy, and steiner tree packing. IEEE Trans. Inf. Theory.

[15] Liu H., Yang J., Wang Y., Chen Y., Koksal C.E. (2014). Group secret key generation via received signal strength: Protocols, achievable rates, and implementation. IEEE Trans. Mob. Comput..

[16] Xiao S., Guo Y., Huang K., Jin L. (2018). Cooperative group secret key generation based on secure network coding. IEEE Commun. Lett..

[17] Tang J., Wen H., Song H.-H., Jiao L., Zeng K. (2022). Sharing secrets via wireless broadcasting: A new efficient physical layer group secret key generation for multiple IoT devices. IEEE Int. Things J..

[18] Ye C., Narayan P. The secret key private key capacity region for three terminals. Proceedings of the 2005 IEEE International Symposium on Information Theory (ISIT).

[19] Zhang H., Lai L., Liang Y., Wang H. (2014). The capacity region of the source-type model for secret key and private key generation. IEEE Trans. Inf. Theory.

[20] Xu P., Yang J., Chen G., Yang Z., Li Y., Win M.Z. (2024). Physical-layer secret and private key generation in wireless relay networks with correlated eavesdropping channels. IEEE Trans. Inf. Forensics Secur..

[21] Ye C., Narayan P. (2012). Secret key and private key constructions for simple multiterminal source models. IEEE Trans. Inf. Theory.

[22] Xu P., Ding Z., Dai X., Karagiannidis G.K. (2016). Simultaneously generating secret and private keys in a cooperative pairwise-independent network. IEEE Trans. Inf. Forensics Secur..

[23] Zhang H., Liang Y., Lai L., Shamai Shitz S. (2017). Multi-key generation over a cellular model with a helper. IEEE Trans. Inf. Theory.

[24] Gong S., Tao X., Li N., Wang H., Han Z. (2020). Secure secret key and private key generation in source-type model with a trusted helper. IEEE Access.

[25] Ahlswede R., Csiszar I. (1993). Common randomness in information theory and cryptography, part I: Secret sharing. IEEE Trans. Inf. Theory.

[26] Maurer U.M., Wolf S. (1999). Unconditionally secure key agreement and the intrinsic conditional information. IEEE Trans. Inf. Theory.

[27] Mao W., Xiong K., Lu Y., Fan P., Ding Z. (2023). Energy consumption minimization in secure multi-antenna UAV-assisted MEC networks with channel uncertainty. IEEE Trans. Wirel. Commun..

